# Development of a High-Density Piezoelectric Micromachined Ultrasonic Transducer Array Based on Patterned Aluminum Nitride Thin Film

**DOI:** 10.3390/mi11060623

**Published:** 2020-06-26

**Authors:** Eunjung Shin, Hong Goo Yeo, Ara Yeon, Changzhu Jin, Wonki Park, Sung-Chul Lee, Hongsoo Choi

**Affiliations:** 1 Department of Robotics Engineering, Daegu Gyeongbuk Institute of Science & Technology (DGIST), Daegu 42988, Korea; ejshin@dgist.ac.kr (E.S.); dusdkfk2@dgist.ac.kr (A.Y.); 2 DGIST-ETH Microrobotics Research Center (DE-MRC), Daegu Gyeongbuk Institute of Science & Technology (DGIST), Daegu 42988, Korea; 3 DGIST Robotics Research Center, Daegu Gyeongbuk Institute of Science & Technology (DGIST), Daegu 42988, Korea; yustchang@dgist.ac.kr; 4 SoC Platform Research Center, Korea Electronics Technology Institute (KETI), Seongnam-si 13509, Korea; wkpark74@keti.re.kr (W.P.); leesc@keti.re.kr (S.-C.L.)

**Keywords:** aluminum nitride, piezoelectric micromachined ultrasonic transducer (pMUT), two- dimensional (2D) array

## Abstract

This study presents the fabrication and characterization of a piezoelectric micromachined ultrasonic transducer (pMUT; radius: 40 µm) using a patterned aluminum nitride (AlN) thin film as the active piezoelectric material. A 20 × 20 array of pMUTs using a 1 µm thick AlN thin film was designed and fabricated on a 2 × 2 mm^2^ footprint for a high fill factor. Based on the electrical impedance and phase of the pMUT array, the electromechanical coefficient was ~1.7% at the average resonant frequency of 2.82 MHz in air. Dynamic displacement of the pMUT surface was characterized by scanning laser Doppler vibrometry. The pressure output while immersed in water was 19.79 kPa when calculated based on the peak displacement at the resonant frequency. The proposed AlN pMUT array has potential applications in biomedical sensing for healthcare, medical imaging, and biometrics.

## 1. Introduction

Compared with conventional bulk ultrasound transducers made with piezoelectric ceramics, micromachined ultrasonic transducers (MUTs) based on thin films can provide more advanced array design, lower power consumption, and better acoustic coupling [1,2,3,4,5]. Notably, MUTs can be manufactured in various shapes and designs, e.g., annular [6], dome-shaped [7], and hexagon-shaped [8] arrays. MUT-based systems integrated with complementary metal-oxide semiconductor (CMOS) circuits are suitable for portable devices. For instance, Lu et al. developed an integrated MUT array with a CMOS using wafer-level conductive eutectic bonding for short-range pulse-echo imaging [9]. An ultrasound fingerprint sensor consisting of an MUT array and CMOS signal processing electronics was demonstrated by Jiang et al. [10]. In the imaging application, the two dimensional (2D) pMUT array can steer and focus ultrasound through electrical delaying signal in 3D, unlike single transducer which needs mechanical drive in order to obtain volumetric ultrasonic imaging [11]. Especially in fingerprint sensing, MUTs provide improved image quality even when there are moisture and contamination on the target surface, unlike a capacitive fingerprint sensor. Capacitive type fingerprint sensor uses electric arrays to recognize the pattern of fingerprint detected by the changes of capacitance between that of the ridge and the valley with air gap from the conductive sensor surface. However, the capacitive fingerprint sensor hardly distinguishes between capacitance of the finger and water [8,9]. According to the operating principle of MUT, the MUT is divided into two types: A capacitive micromachined ultrasonic transducer (cMUT) and a piezoelectric micromachined ultrasonic transducer (pMUT).

The cMUT has a cavity sandwiched between the top and bottom electrodes which require fewer layers leading to simple fabrication. To produce an ultrasound beam, the deflection of the top electrode membrane is achieved electrostatically. However, this cMUT, which requires a high DC bias voltage (>100 V), has limited applications because of safety issues [5,12]. Recently, a cMUT using a low input voltage has been developed [13], but still the DC bias limits the portability of the cMUT array for the biometric application. In contrast, the pMUT consisting of multi layers such as piezoelectric thin film and electrode layers fabricated through complexity steps produces acoustic waves under applied low alternating voltage without high bias [2,14,15,16]. Thus, pMUTs are suitable for bio-applications including medical imaging [17], stimulation [18], and fingerprint sensing [11].

In terms of the pMUT vibration mechanism, out-of-plane deflection of pMUTs is caused by in-plane stress in the piezoelectric layer when an electric field is alternately applied between the top and bottom electrodes. This relationship is represented by the transverse piezoelectric coefficient. The sensing sensitivity ($G_{S}$) of a pMUT is proportional to the effective transverse piezoelectric coefficient ($e_{31,\;f}$) and inversely proportional to the dielectric constant ($\varepsilon_{33}$), as follows [2]:

(1) $$G_{S}\propto e_{31,\;f}/\varepsilon_{33}$$

Lead zirconate titanate (PZT) [18], zinc oxide (ZnO) [19], and aluminum nitride (AlN) [20,21,22,23] are widely used for piezoelectric layers to govern the performance of pMUTs. The lead-free AlN, having high mechanical hardness [24] and allowing to maintain piezoelectric properties at high temperatures (<900 °C), is a good candidate for a pMUT [25,26]. Notably, according to Equation (1), AlN has 10-times higher sensing sensitivity ($e_{31,\;f}$ = 1.08 C/m^2^, $\varepsilon_{33}$ = 10.5) than PZT ($e_{31,f}$ = −14 C/m^2^, $\varepsilon_{33}$ = 1200) [27] due to its low dielectric constant. The piezoelectric coefficient is intimately linked to the crystal orientation of AlN [28]. Therefore, well-oriented AlN, which improves the piezoelectric coefficient, needs to be used in a pMUT for sensing devices.

Herein, we propose a d_31_ mode compact 2D pMUT array based on an AlN thin film. As for considering the restriction of fabrication process including membrane releasing, the 20 × 20 pMUT elements with small radius (40 µm) and pitch (20 µm) were proposed and built on a 2 × 2 mm^2^ area to improve fill factor (device area/membrane area). The membrane with AlN thin film was designed with a circular shape, which has high efficiency due to small grating lobes according to the previous finite element method (FEM) studies [2,14,29,30]. Bump structures consisting of a gold (Au)–tin (Sn) multilayer were introduced onto four top electrode pads of each pMUT to enable future integration with a CMOS chip [31,32,33]. Integration with CMOS allows for a large number of pMUT elements with improved amplification, high signal strength, low power consumption, and compact size [34]. The microstructure and crystallinity of the AlN were analyzed to evaluate the quality of the thin film. The vibration characteristics of the structures of the proposed AlN pMUT array were evaluated based on the impedance spectrum and surface mechanical displacement data.

## 2. Materials and Methods

**Figure 1:**
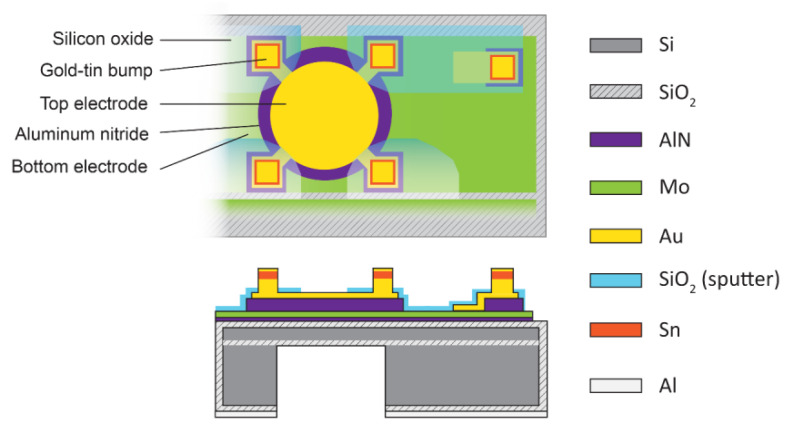
Top view and cross-sectional schematic illustrations of an AlN pMUT element.

**Figure 2:**
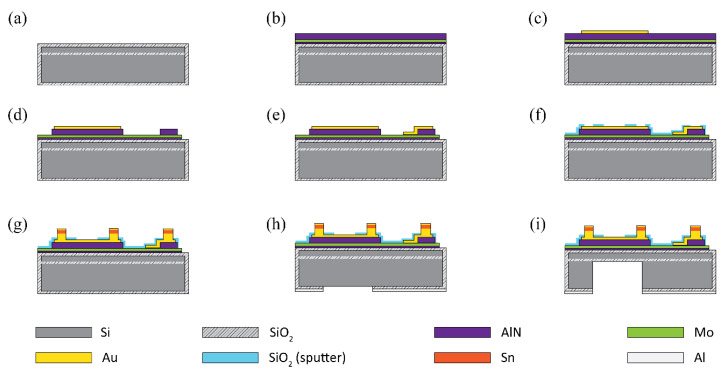
Fabrication process of the AlN pMUT array; (**a**) Diffusion of a silicon oxide layer. (**b**) Deposition of seed layer (AlN), bottom electrode (Mo) and AlN piezoelectric layer. (**c**) Deposition and patterning of top electrode (Au). (**d**) Patterning of AlN layer. (**e**) Deposition and patterning of connecting layer (Au) for bottom electrode. (**f**) Deposition and patterning of silicon oxide layer. (**g**) Deposition and patterning of Au-Sn bumps. (**h**) Patterning of silicon oxide using hard mask (Al) on backside. (**i**) Deep silicon etching on backside to release the membrane.

**Figure 3:**
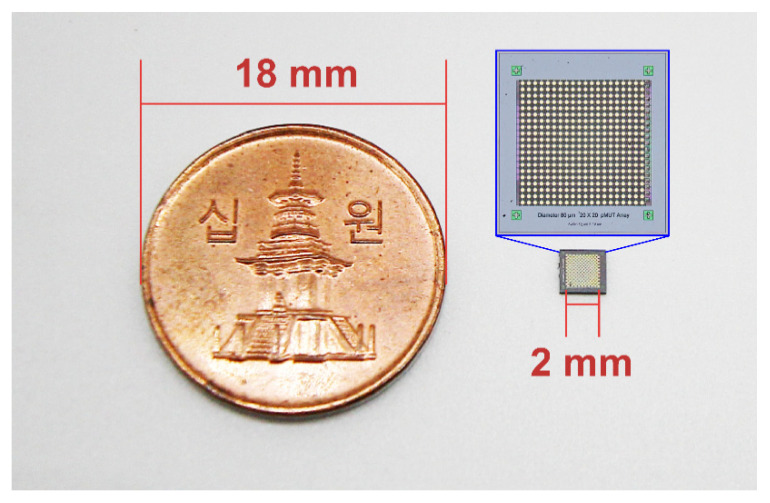
Photograph of the fabricated 20 × 20 AlN pMUT array (400 elements) beside a 10-won Korean coin.

Figure 1 shows the top view and cross-sectional schematic illustrations of the designed pMUT element in the 20 × 20 array. Each pMUT element has a 28-µm-radius circular top electrode (Au) with four square bumps. The 20 pMUTs in each row share rectangular molybdenum (Mo) bottom electrode with one Au–Sn eutectic bump on the AlN pillar that provides mechanical support and electrical contact. The circular-patterned AlN thin film has a larger area (5024 µm^2^) than the top electrode (2461 µm^2^) to prevent an electrical short between the top and bottom electrodes. Based on the design of the pMUT shown in Figure 1, a mm-sized AlN pMUT array was fabricated using the process presented in Figure 2. A 6-inch silicon-on-insulator (SOI) wafer containing a 5-µm-thick silicon layer and 1-µm-thick buried oxide layer was used to make the pMUT array. First, a 200-nm-thick silicon dioxide (SiO_2_) layer for electrical insulation was formed on the silicon wafer surface via diffusion of oxygen using a high-temperature diffusion furnace (KVD206; KSM Component, Cheongju, Korea) (Figure 2a). A 50-nm-thick AlN film was deposited on the SiO_2_ surface as a seed layer, and a 200-nm-thick Mo layer was used as the bottom electrode to achieve the preferred c-axis orientation of the AlN layer [35,36]. Then, a 1-µm-thick AlN thin film grown by radio frequency (RF) magnetron sputtering was used as a piezoelectric layer (AMS, CA, USA) (Figure 2b). A 200-nm-thick Au top electrode film was sputtered at a chamber pressure of 6 mTorr of argon (Ar) gas (150 sccm) (SRN-110; Sorona, Anseong, Korea) and patterned by the lift-off method using a 1.7-µm-thick photoresist (AZ GXR 601 46 CP; Merck KGaA, Darmstadt, Germany) (Figure 2c). Then, patterned AlN films were obtained by etching process using a plasma etcher (FabStar; Top Technology Limited, Hwaseong, Korea) in a mixture of boron trichloride (BCl_3_), chlorine (Cl_2_), and Ar gases. The Mo layer was etched under the same conditions as for the AlN layer to achieve a long rectangular shape and allow the same bottom electrode to be shared by each row of elements (Figure 2d). To link the bottom electrode and eutectic bump on the square-patterned AlN layer, a 200-nm-thick Au layer was sputtered and patterned using the lift-off technique (Figure 2e). Prior to forming the Au–Sn layers, a 200-nm-thick patterned SiO_2_ layer was deposited to prevent the possibility of a short between the top and bottom electrodes during the bonding process (Figure 2f). Square-shaped Au–Sn bumps (20 × 20 µm^2^) were deposited on the electrode pads and AlN pillar before the backside silicon etching process. Au–Sn multilayers display good stability and thermal cycling ability, and excellent fatigue resistance when used for eutectic bonding of integrated circuits [33,34]. To achieve the eutectic composition of Au and Sn (80 and 20 wt%, respectively) [33], a 10-nm-thick titanium (Ti) adhesion layer, 800-nm-thick Au layer, 300-nm-thick Sn layer, and 20-nm-thick Au cap passivation layer were continuously sputtered without breaking the vacuum using a multitarget RF magnetron sputter system. The stack of bump structures was patterned into the 15 × 15 µm^2^ square shape using the lift-off method (Figure 2g). Finally, the backside of the silicon wafer was etched with circular patterns using deep reactive ion etching (DRIE) (LPX PEGASUS; SPTS, Newport, UK) to release the device membrane. For this step, the 200 nm thick SiO_2_ layer on the backside of the wafer was removed by wet etching using buffered oxide etchant (BOE) while the top surface of wafer was protected with 8 µm thick photoresist (AZ9260; Merck KGaA, Germany). A 300-nm-thick aluminum (Al) layer was sputtered and patterned as a hard mask for the backside silicon etching (Figure 2h). Then, high-aspect-ratio anisotropic DRIE silicon etching was performed based on the Bosch process. This process involved silicon etching and surface passivation using sulfur hexafluoride (SF_6_) and octafluorocyclobutane (C_4_F_8_) gases, respectively [37,38]. The 650-µm-thick silicon layer was etched to the buried oxide layer at a rate of 8 µm/min. The through-wafer silicon etching is crucial to achieving the desired membrane dimensions and the resonant frequency of the pMUT device. Fabrication of the 40-µm-radius pMUT array was accomplished via through-wafer DRIE (Figure 2i). Figure 3 presents the final 20 × 20 pMUT array.

**Figure 4:**
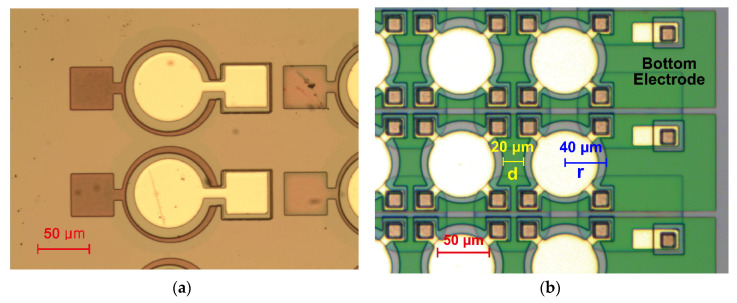
Fabricated (**a**) single-element and (**b**) 20 × 20 array of AlN pMUTs.

Figure 4a,b show top-view microscope images of the fabricated pMUT devices having single and 2D array designs, respectively. The single AlN pMUT element (radius: 40 µm) had 40 × 40 µm^2^ top and bottom electrode pads. The pMUT elements in the 2D array had four 20 × 20 µm^2^ top electrode pads with bumps, which shared a bottom electrode (the green-shaded region in Figure 4b). The 45 × 20 µm^2^ rectangular-shaped Au patterns connected the eutectic bumps and bottom electrode near the right edge of each row. The pitch (d) and radius (r) of the pMUT elements were 20 and 40 µm, respectively (Figure 4b). The small radius and pitch between the pMUT elements led to a high fill factor of 50.27% (${\pi r}^{2}/{\left(2r+d\right)}^{2})$, which ensured high-pressure output and sensitivity [11,39]. 

## 3. Results and Discussion

**Figure 5:**
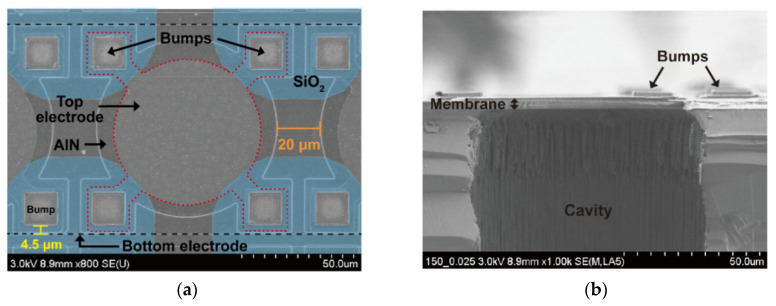
Scanning electron microscope images of the fabricated AlN pMUT array. (**a**) Top view and (**b**) cross-sectional view.

Surface and cross-sectional images of the pMUT elements were obtained by scanning electron microscopy (SEM; SU8230; Hitachi, Tokyo, Japan). Figure 5a,b clearly reveal the geometric structure of the pMUT element and the released diaphragm. The top electrode, indicated by the red dashed line, has a circular pattern with Au–Sn bumps on the four corner pads. Using four-bump structures instead of pairing an element with a single bump could enhance the binding strength with the circuit chip through eutectic bonding. There was a risk that the short distance (4.5 µm; yellow bar) between the bump and the bottom electrode could cause an electrical short due to over melting of the Au–Sn bumps during the eutectic bonding process (Figure 5a). Thus, SiO_2_ passive layers were located around the bumps to prevent this possibility. The relationship between pMUT performance and device geometric parameters, such as the top electrode coverage, has been validated [2,22,40]. Based on experimental and theoretical analyses, the radius of the top electrode was set to 70% of that of the membrane. Previous work established that this size maximized the center deflection of the membrane, and the effective electromechanical coupling coefficient ($k_{eff}^{2}$) [40]. The pitch of the pMUT elements was 20 µm (orange bar in Figure 5a). Compared with previous researches such as Chen et al. (25%) [19] and Jiang et al. (51.7%) [11] the proposed AlN pMUT array has relatively high fill factor (50.3%) which could lead the high acoustic pressure. Figure 5b shows the AlN membrane sandwiched between the bottom and top electrodes built on the supporting silicon membrane over the deeply etched silicon cavity.

**Figure 6:**
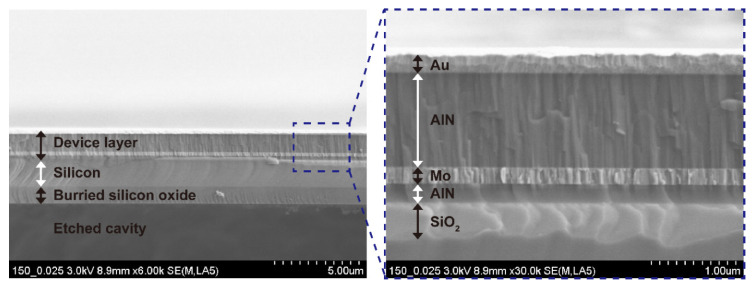
Cross-sectional scanning electron microscope images of the membrane structure (left image) and magnified device multilayer (right image) of the AlN pMUT element.

**Figure 7:**
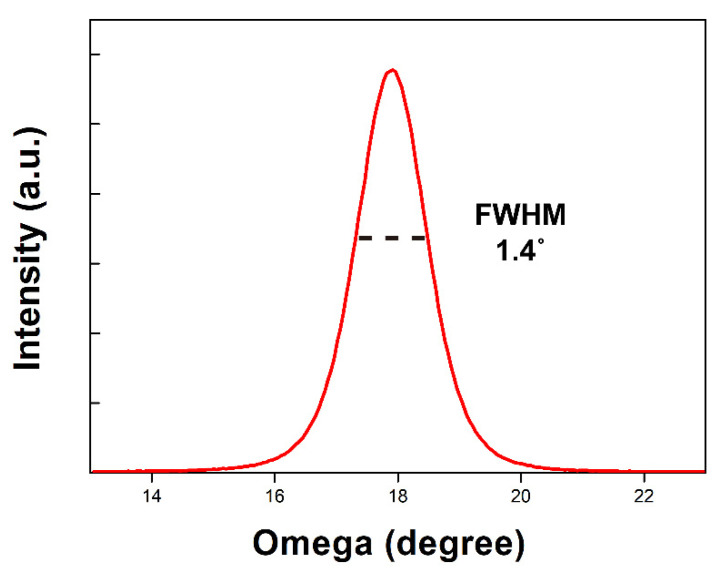
A rocking curve of the sputtered AlN film.

The cross-sectional SEM image in Figure 6 shows the multilayer consisting of the AlN seed layer, Mo bottom electrode, AlN active layer, and Au top electrode above the cavity. Vertical columnar crystallites with dense structures are evident in the 1-µm-thick AlN layer shown in the magnified image. The crystallinity of the sputtered AlN thin film was characterized by X-ray diffraction (XRD; Empyrean; Malvern Panalytical, Malvern, UK). Figure 7 shows the rocking curve of the AlN thin film at the (0002) peak position. The full-width at half-maximum (FWHM) was 1.4°, which indicated highly oriented crystallinity and guaranteed good piezoelectric properties of the sputtered AlN film [26].

**Figure 8:**
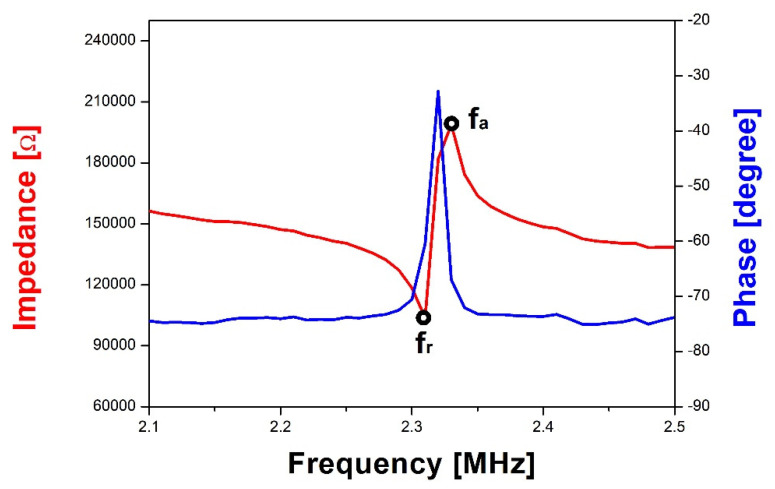
Impedance and phase of an AlN pMUT element of the 20 × 20 array.

For the electrical characterization of the pMUT, the impedance frequency spectrum of the fabricated pMUT array was measured using an impedance analyzer (HP4294A; Agilent Technology, Santa Clara, CA, USA) at 500 mV oscillation (Figure 8) at room temperature (RT). The impedance showed minimum and maximum values at the resonant and anti-resonant frequency, respectively. The average resonant frequency of five AlN pMUTs in the 20 × 20 array was 2.82 MHz and the standard deviation was 14.1%. The deviation in resonant frequency is substantially expanded by the DRIE process variation [2]. In addition, a residual stress on membrane may additionally increase the variation of resonant frequency as compared with Robichaud et al. [41]. The effective coupling coefficient, defined as the conversion ratio between the electrical energy and the mechanical energy, is generally extracted from electrical impedance measurements as follows [6]:

(2) $$k_{eff}^{2}=\frac{f_{a}^{2}-f_{r}^{2}}{f_{a}^{2}}\;≅\;\frac{C_{m}}{C_{0}+C_{m}}$$

where $f_{a}$*,* $f_{r}$*,* $C_{m}$, and $C_{0}$ are the anti-resonant frequency, resonant frequency, motional capacitance, and passive capacitance, respectively. A high effective coupling coefficient indicates large mechanical and electrical bandwidths, which ensure high resolution for ultrasonic imaging [2]. The average effective electromechanical coupling coefficient of five fabricated pMUT elements in the array was 1.1%.

**Figure 9:**
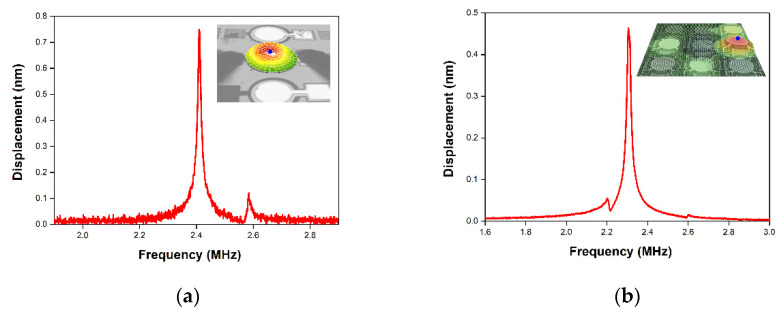
Mechanical displacement of the fabricated AlN pMUT device. (**a**) Single and (**b**) array pMUT with periodic chirp input.

**Figure 10:**
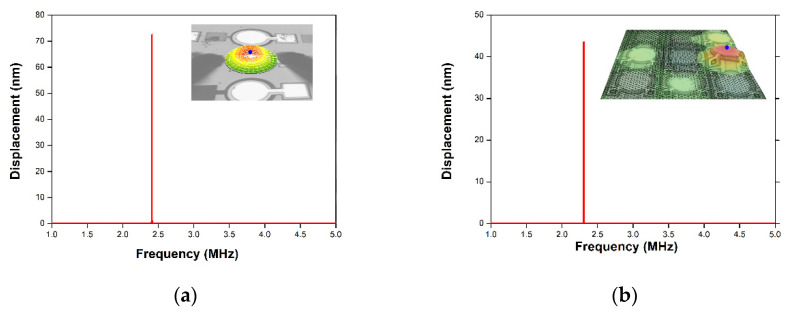
Mechanical displacement of the fabricated AlN pMUT device. (**a**) Single and (**b**) array pMUT with sinusoidal input.

The dynamic performance of the pMUT devices was determined by laser Doppler vibrometry (LDV; MSA-500; Polytec, Germany) at RT under the air atmosphere. Figure 9 presents the mechanical displacement as a function of frequency (1.0–5.0 MHz) during the application of a 3 V periodic chirp signal. The center mechanical displacements of a single pMUT and 2D array were 748 and 451 pm, respectively. And the quality factor of single AlN pMUT and array was calculated from displacement results as 111.9 and 73.7 respectively. The resonant frequency of the selected pMUT element were determined from the mechanical displacement results in periodic chirp input. In the sinusoidal input measurement, the electrical input signal was applied at the resonant frequency of the selected pMUT element in array. When a 3 V sinusoidal input voltage was applied to the single pMUT and the selected element pMUT in array at the resonant frequency of 2.41 MHz and 2.31 MHz respectively, the mechanical peak displacement increased by 72.4 and 43.4 nm as shown in Figure 10. The transmitting sensitivity of selected pMUT in array is 14.5 nm/V which is a key parameter to effect output pressure. The five neighboring pMUTs surrounding the activated pMUT in closed packed array exhibit small mechanical amplitude around 2.82 nm (6% of mechanical displacement of the selected pMUT) caused by inevitable acoustic interaction. However, this amplitude level by the crosstalk effect does not seem severe due to the patterned AlN thin film [9].

The acoustic performance of the pMUT was evaluated based on the acoustic pressure of the pMUT array generated by the membrane oscillation, as follows [11]:

(3) $$P=\left(2\pi f_{r}d_{P}\right)Z_{a}A_{eff}\sqrt{F}$$

where $Z_{a}$ is the acoustic impedance, $d_{P}$ is the maximum displacement at the membrane center, $A_{eff}$ is the effective area, and F is the fill factor of the pMUT array. According to Equation (3), the surface pressure P is linearly proportional to the frequency, displacement, and diameter of the membrane. The resonant frequency and mechanical displacement of the fabricated AlN pMUT in water were measured using the LDV system. The AlN pMUT was immersed in a dish filled with 1 cm of deionized water and a 10 V sinusoidal signal was applied via microprobes. The average resonant frequency was 1.47 MHz, and the mechanical displacement at the center point was 54.88 nm (transmitting sensitivity = 5.5 nm/V). The pMUT immersed in water exhibited a lower transmitting sensitivity and resonant frequency than in air due to the dampening effect of the water, which has a greater viscosity $\left(1.0\times 10^{-3}\;\mathrm{kg}/m\cdot s\right)$ than air $\left(1.983\times 10^{-5}\;\mathrm{kg}/m\cdot s\right)$ [42]. The acoustic pressure of the fabricated pMUT array with the high fill factor was estimated as 19.79 kPa, which is comparable to previously reported AlN pMUT sensors developed by Wang et al. as 6 kPa [32], Jiang et al. as 15 kPa [11], and Liu et al. as 1 kPa [20] at their resonant frequency in water, even at relatively low resonant frequencies [9,18,30].

## 4. Conclusions

A 2D array of pMUTs based on a strongly c-axis-oriented AlN thin film was designed, fabricated, and characterized. The deposition and patterning of the AlN thin film were done by sputtering and plasma etching with lithography. Miniaturized pMUTs (N = 400, each 40 µm in radius) were integrated into a 2 × 2 mm^2^ area and displayed a high fill factor of 50.27%. The pMUT membrane having a 20 µm pitch was successfully released by anisotropic silicon etching via the DRIE process. The average resonant frequency of the fabricated pMUT array was 2.82 MHz in air. Impedance and phase measurement results established a maximum electromechanical coefficient of 1.7% for the pMUT. The output pressure of the 20 × 20 pMUT array in water was 19.79 kPa when the mechanical displacement was 54.88 nm with a 10 V sinusoidal input. Furthermore, the four Au–Sn bumps introduced on each top electrode allow integration with a CMOS chip in a miniaturized ultrasonic sensing system. The demonstrated AlN-based high fill factor pMUT array shows great promise for portable and implantable imaging applications.
